# Verbal or Written? The Impact of Apology on the Repair of Trust: Based on Competence- vs. Integrity-Based Trust Violation

**DOI:** 10.3389/fpsyg.2022.884867

**Published:** 2022-04-25

**Authors:** Shuhong Gao, Jinzhe Yan

**Affiliations:** ^1^School of International Education, Changzhou Vocational Institute of Mechatronic Technology, Changzhou, China; ^2^School of Business, Gachon University, Seongnam-si, South Korea

**Keywords:** verbal apology, written apology, trust repair, competence-based trust violation, integrity-based trust violation

## Abstract

This study examined the effect of verbal and written apologies on trust repair based on competence and integrity after a trust violation. Through three experiments, the empirical results showed that the written apology was more effective than verbal ones a restoring trust for integrity-based trust violations. However, the verbal apology was more effective against competency-based trust violations than a written one. Moreover, the results also showed that perceived trustworthiness played a mediating role between trust violation and trust repair, while positive emotions played a moderating role. Finally, this study provided a general discussion, implications, and suggestions for future research.

## Introduction

The positive effect of trust on individuals, teams, and organizations has been widely confirmed by empirical research (Colquitt et al., 2007). Trust can reduce consumers’ sense of insecurity, improve their satisfaction and loyalty, and maintain a good and sustainable relationship (Palvia, 2009). However, trust is fragile and can be easily damaged or destroyed (Kramer, 1999; Kim et al., 2013). Compared with the positive information that enhances trust, the negative information that destroys trust is more likely to attract the sender’s attention. In the process of trustworthiness judgment and evaluation, negative information occupies a higher decision-making weight than positive information. Because of this typical asymmetry, the development, destruction, and decline of trust have become the norm (Slovic, 1993).

How can trust be repaired and rebuilt after it has been broken? The researchers studied apology, denial, silence, explanation, commitment, justification, voluntary collateral, compensation, punishment, and other verbal responses repair strategies (Bottom et al., 2002; Nakayachi and Watabe, 2005; Schweitzer et al., 2006; Tomlinson and Mryer, 2009). Among these strategies, the research on apology mainly focused on the content and function of apology. Apologies can positively influence the trustor’s assessment of the trustor’s motivation, reduce the trustor’s fear of future harm, and eventually improve the trustor’s level of trust (Blackman and Stubbs, 2001). Although apologies can be divided into verbal and written apologies, existing research has not solved which form of apology is more sincere and compelling.

The type of trust violation is also a hot topic for academic research. The most critical is to analyze how trust is broken because different ways of breaking trust may require different remedial measures (Schoorman et al., 2007). Kim et al. (2004) classified the types of trust violations into competence-based violations and integrity-based violations. Kim et al. (2006) concluded that if an external attribution apology is made for integrity-based violations, the trust would be better repaired; on the contrary, an internal attribution apology would be better repaired for competency-based violations. However, Kim et al. (2006) did not consider the form of apologies in their research. In the practice, enterprises or individuals often use verbal or written forms of apology in the process of apology, but their effectiveness has not been thoroughly studied. Therefore, as a result, there has been no research on whether verbal or written apologies are more effective.

Continuity of relationships stems from the trust, and Mayer et al. (1995) put forward the accepted credibility in terms of competence, kindness, and integrity. When faced with violation behavior (such as betrayal, violation, deception), an individual’s perception of the offender might be damaged, and credibility might be reduced (Kim et al., 2004).

The researchers found that trust was not a rational cognitive process, and emotional factors affect trust evaluation. Scholars generally believe that trust consists of emotional, cognitive, and behavioral components, and trust is divided into cognitive trust and emotional trust (Weber and Carter, 2003; Lewis and Weigert, 2012). Therefore, emotional expression is inevitable and crucial in the process of trust repair. In recent years, with the rise of positive emotion research, many scholars have found that positive emotion has a significant effect on promoting interpersonal trust, thus establishing a link between emotion and trust, positive emotion and trust repair, and other research fields (Lount, 2010). Therefore, when transgression involves integrity and competence, how do positive emotions play a role?

This research has three main contributions. Firstly, this study expands the literature on apology types, including verbal and written ones, and examines the validity of trust violations responses. Secondly, researchers have studied the relationship between perceived trustworthiness and apology and believe that apology improves the perceived credibility of offenders, thus increasing trusting behavior (Ma et al., 2019). Therefore, we examine the relationship of perception credibility between violation and trust repair. Thirdly, in view of the influence of the promotion of existing positive emotions on interpersonal trust, this study attempts to expand the scope of research, namely, the moderating effect of positive emotions on trust violation and trust repair and explore the influence of positive emotions on consumers’ trust repair in the situation of competence and integrity trust violation.

## Theoretical Background and Hypotheses Development

### Trust Formation, Violation, and Repair

#### Trust Violation and Repair

This study defines trust as the psychological state of being willing to accept a weak position based on positive expectations of the intentions and actions of others (Rousseau et al., 1998). Following (Mcknight et al., 1998), Kim et al. (2006) identified two factors of trust: trusting intentions and trusting beliefs. A violation of trust occurred when the trusting party perceived that the trusted party’s behavior did not conform to its expectations. Trust violation refers to the phenomenon of trust damage caused by the behavior of the violator not meeting the positive expectations of the victim. This study recognizes that trust structure is very complex, and trust repair needs to focus on trust beliefs and trust intentions. Therefore, we believe that trust repair is activities that try to make trust intention and trust belief more positive after a trust violation is felt.

#### Apology

After a violation of trust, a formal apology may be a prerequisite for restoring a trusting relationship. By apologizing, the offender acknowledges the harm done to the victim, expresses remorse and reconciliation, and hopes to continue to maintain a good relationship (Goffman, 1972; Tedeschi and Norman, 1985; Lewicki and Bunker, 1996). It is an important step to reduce mistrust after violations, as it conveys to the injured a recognition of injustice and a desire to restore fairness (Greenberg, 1990). In addition, apologies can represent an influential social account and help victims get more information about violations and the exact nature of the offender. For example, Greenberg (1990) describes the apology as “an attempt to convince the audience that any attribution made on the basis of an actor taking responsibility for an undesirable event is inaccurate.” Research shows that apologies are essential for reducing aggression in injured people and that more severe aggression requires more extensive apologies (Ohbuchi et al., 1989; Sitkin and Bies, 1993).

Tomlinson et al. (2004) divided apology into three categories: one was the “no-apology” response, and there was no explicit apology to the victim; The second kind thought that internal factors caused the mistake. The apology of internal attribution admitted that the mistake was caused by one’s own weakness (such as ability). The third category attributed the conflict to external factors. Apologies for external attribution assumed that external factors contributed to the mistake (for example, circumstances). An apology with internal attributions may be more effective in motivating reconciliation because the offender takes responsibility instead of passing it off. Conversely, those who shift the blame to external factors, although they seem to have no responsibility, their reputation may be affected (Schlenker et al., 2001).

The violation of trust often causes negative emotions in consumers, such as sadness and anger. Apology expresses care, sincerity, politeness, and empathy to consumers to reduce their negative emotions and alleviate their feelings of injustice. Therefore, apology is considered to be an effective measure to restore trust (Smith et al., 1999; Tomlinson and Mryer, 2009). However, some researchers argue that apologies, because they admit guilt, may not ameliorate the negative consequences of trust violation (Riordan et al., 1983). Therefore, on the one hand, an apology expresses repentance and indicates the intention not to repeat such violations in the future, thus restoring trust; on the other hand, an apology acknowledges guilt and indicates that the violator should be blamed, which may reduce trust (Kim et al., 2006). This contradiction has prompted researchers to expand the field of study to types of apologies. Although they admit guilt and hope to avoid a violation, which one reduces trust, and which one restores it?

Tomlinson et al. (2004) proved that apologies were more effective than no apologies in trust repair, and apologies with an internal attribution are more effective than apologies that blame the behavior of broken promises on external reasons. However, other studies have shown that this conclusion has limitations. For example, in the context of different types of trust violations, Kim et al. (2006) demonstrated that external attribution apologies repair trust better than internal attribution apologies for integrity-based trust violations. However, if there is a violation of competency-based trust, an internal rather than external attribution apology should be used. This is because when trust violations involve integrity, people tend to focus on the negative information about integrity. Furthermore, they tend to value positive information when trust violation involves competence (Snyder and Stukas, 1999).

According to the different forms of apology, it can be divided into written apology and verbal apology. The written apology is more formal and solemn and can reflect sincerity, usually with the apology letter as the carrier, easy to retain, is a relatively stable, long-term, and solidified comfort to the victim. On the other hand, the verbal apology is through the verbal language to apologize. However, sincerity is not as good as a written apology, but “face to face” apology, through language, tone, eyes can highlight the apology’s ability, enabling people to feel the spiritual comfort and convenient, flexible operation directly (Zhang, 1996).

#### Violation Type

Which is more effective, a verbal apology or a written apology? Whether a violation of trust involves competence or integrity issues may be a factor that plays a key role. Ferrin et al. (2007) and Kim et al. (2006) analyzed the effect of apology and other verbal responses on trust repair through a series of studies. They found that the type of trust violation affected the effectiveness of the apology. They represent the two most important qualities in determining credibility (Cook and Wall, 1980; Barber, 1983; Butler and Cantrell, 1984; Schindler and Thomas, 1993; Mayer et al., 1995). Two kinds of expectations were thought to involve some basic implications of trust: the performance expectation of technically competent roles, and the other is the expectation of the continuity and fulfillment of natural and moral social orders (Barber, 1983). This concept was supported by empirical evidence (Butler and Cantrell, 1984).

Moreover, prior research has shown that these dimensions offer essential bases on which individuals evaluate a variety of targets, such as potential collaborators (Kee and Knox, 1970). Butler and Cantrell (1984) defined competency-based trust as the principal’s belief that the trustee has the technical and interpersonal skills required for the job. Mayer et al. (1995) defined integrity-based trust as a set of principles that the principal considers acceptable for the trustee to abide by.

According to the attribution bias theory of Reeder and Brewer (1979), from the perspective of competence, individuals attach more importance to positive information because it is intuitively assumed that high-competence individuals are capable of exhibiting performance at many levels based on motivation and task demands. Conversely, those with low competence can only perform at levels commensurate with or below their ability level. From the perspective of integrity, individuals value negative information more because people intuitively believe that honest people will not behave dishonestly under any circumstances while dishonest people may behave dishonestly or honestly, depending on their motivation and opportunities (Kim et al., 2006).

In summary, active verbal apology, combined with language, eyes, and body movements, for a competency-based trust breach can soothe people emotionally, reflect their abilities, meet people’s expectations, and repair trust (Ma, 2006). Comparatively speaking, the written apology only through words, reflecting the lack of repair effect, is not as good as a verbal apology. For a violation of trust based on integrity, being sincere provides a strong signal that honest behavior is coming. Written apologies are more formal, solemn, and can be kept long. They are a long-term commitment with an engagement and can repair trust better than verbal ones.

H_1a_: Apology type positively moderates the relationship between trust violation and trust repair.

H_1b_: When trust violation involves competence, trust may be better repaired by responding with a verbal apology rather than a written one.

H_1c_: When trust violation involves integrity, trust will be better repaired by responding with a written apology rather than a verbal one.

### Perceived Credibility

Politicians, orators, and public speakers have attempted to identify the determinant characteristics of effective speakers (Giffin, 1967). Likewise, previous research attempts to determine the components of source credibility.

If the source of information is credible, it is valid. Aristotle defined credibility as the quality of a source of information that is the most credible of all evidence. Credibility plays a decisive role in determining the validity of an endorsement (Amos et al., 2008). If a source is credible, it helps to have a more positive attitude toward advertising (Muda et al., 2014). Moreover, credible sources will also affect consumers’ purchase intentions (Lafferty et al., 2002). Verma and Kapoor (2004) found that many participants admitted to buying a product only because they admired the particular celebrity who endorsed the product. Hovland Carl et al. (1953) pointed out that two factors lead to celebrities’ perceived trustworthiness: experts and trustworthiness. Credibility refers to the degree of trust and acceptance of the speaker and the information conveyed by the audience (Hovland Carl et al., 1953). Gaziano and Mcgrath (1986) found that source credibility included security, qualification, and vitality dimensions. Ohanian (1990) proposed constructing a multidimensional credibility measure, which included three dimensions: attractiveness, expertise, and credibility. Newell and Goldsmith (2001) conducted five studies to purify two scales, one on the credibility aspect of credibility and the other on the professional aspect.

Previous studies suggested that the act of an apology could reduce the negative impression of violators (Darby and Schlenker, 1982, 1989) and indicate trustworthy intentions and tendencies in the future (Schniter and Sheremeta, 2014). Apologizing could improve the offender’s trust and increase the trust behavior (Ma et al., 2019). When trust violation occurs, the perceived credibility of the victim will be reduced, and trust will be destroyed.

When trust violation involved competence, people would think that the offender was not competent, and those with low competence could only act following or below their competence level (Reeder and Brewer, 1979). However, the improvement of competence was not achieved overnight and cannot be promoted in a short period. These factors would affect the judgment of credibility, which affected trust and repair. When trust violation involves integrity, people with low integrity might exhibit dishonest or honest behavior, depending on their motivations and opportunities (Kim et al., 2006).

H_2a_: Perceived credibility mediates the relationship between trust violations and trust repair.

H_2b_: Perceived credibility has a more significant impact on repairing trust under competence-based violations than integrity-based ones.

### Positive Emotion

Positive emotion is a kind of joyful feeling, which is the joyful experience of individuals when their needs are met, goals are achieved, or things are going well (Russell and Barrett, 1999). According to the positive emotion expansion and construction theory, positive emotion was a temporary pleasure, an individual’s unique and immediate response to meaningful things. Positive emotions enhanced the cognitive domain (Fredrickson, 2003). Positive emotions were related to the behavioral approach and were also the accompanying emotional reactions in the process of the behavioral approach (Davidson et al., 1990). Frijda (1986) documented those positive emotions should include happiness, interest, desire, and wonder. In addition, Lazarus (1991) believed that happiness, pride, hope and love were positive emotions. Ekman (1992) supposed that positive emotions included joy and surprise, while Fredrickson (1998) indicated that positive emotions include joy, interest, contentment, and love. Therefore, happiness and cheerfulness were essential indicators of positive emotions.

Wyer (1979) believed that emotional states had informational and direct functions in information processing. According to the affect-as-information perspective, individuals tended to consider their feelings about the target rather than made judgments by measuring other factors. Moreover, their reactions to the target were based on the emotional states they have experienced before making judgments in the process of making a judgment (Sarwar et al., 2013). Lount (2010) found that when other groups had target cues (credibility) that promoted interpersonal trust, a positive mood enhanced interpersonal trust, indicating that target cues influenced the cognitive processing of emotional, interpersonal trust.

Positive emotions increased the predictability of the offender’s behavior and indicated that the environment is safe and reliable, increasing trust. Studies have shown that even the accompanying emotions unrelated to the trust situation can impact trust, and positive emotions such as happiness can enhance trust (Dunn and Schweitzer, 2005). For example, when trust violation involves integrity, positive emotions can make the victim feel that honesty is coming, which can better eliminate the negative effects of trust violation. On the other hand, when the violation involves competence, the offender is considered incompetent, and competence is less affected by situational factors. Therefore, the repair effect of positive emotion is worse than that of integrity violation.

H_3a_: Positive emotions moderate the relationship between trust violations and trust repair.

H_3b_: Positive emotions have a greater impact on repairing trust under integrity-based trust violations than competence-based trust violations.

**FIGURE 1:**
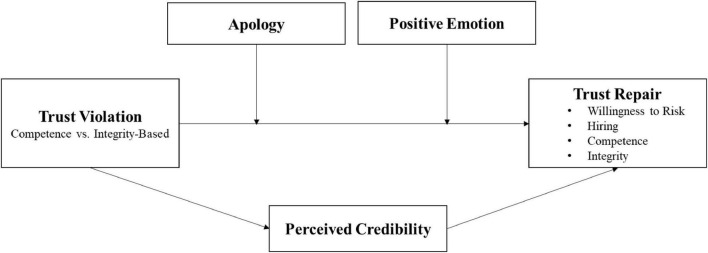
Proposed research model.

Our proposed framework is shown in Figure 1.

## Experiment Design, Procedure, and Statistics Analysis

### Study 1

To investigate the moderating effect of apology and the effect of Verbal versus written apology, in study 1, we extended the laboratory experiment developed by Kim et al. (2004). In Kim’s study, participants watched a video of an interview for a senior-level tax accountant, and at the end of the video there was a violation by the candidate. Finally, participants completed a questionnaire. We asked participants to read the materials about the employment interview, in which the candidates responded with verbal and written apologies for violating the trust based on competence or integrity in their previous job. After reading the material, participants were asked to report their trust in the applicant. This study implemented a 2 (type of violation: competence vs. integrity) × 2 (verbal vs. written apology) between-subjects design.

#### Method

Participants. Four hundred ninety-nine students from a management course at a college in eastern China took part in the study as part of a classroom exercise. The average age of the participants was 20 and 78% were male.

Task. The role of the participant was set as a hiring manager, responsible for recruiting and managing a senior tax accountant. If the candidate was hired, he/she would receive a one-year contract, subject to renewal based on an annual performance review. First, managers read a piece of written material and then evaluate the candidate.

Manipulations. According to our 2 × 2 between subject’s design, the written materials include the basic situation of the candidate’s interview. The end of the material shows the candidate’s accounting-related violations in his/her previous work. The framework and response to such violations of trust represent research operations.

Violation type. We classify a violation of trust as an integrity-based or a competency-based one. We set up a situation where the recruiter told the candidate that he/she had contacted the candidate’s former employer and learned that the candidate had behaved inappropriately at his/her previous job. For example, deliberately understating a client’s taxable income in the integrity condition. In the condition of competence, the applicant was accused of filing an incorrect tax return due to an insufficient understanding of tax laws. In two cases, the information was anecdotal; The recruiter had no hard evidence to prove the truth of the allegations.

Apology. Immediately after mentioning the violation, the candidate attempted to restore trust by taking responsibility for the relevant behavior through a verbal or written apology. With a verbal apology, the candidate admits his/her bad behavior and takes full responsibility for it. He/she also promises that he/she will never do it again. Finally, he/she promises not to have any concerns about his/her integrity/competence if the company hires him/her. The applicant admitted the violation and apologized in a written letter in a written apology.

Manipulation checks. We designed three questions to assess whether participants were aware that they had been assigned to different experimental conditions. The first two questions assess whether they recognize the nature of the violation differently. All participants answered three operational inspection questions. Specifically, respondents were first asked, “In the material, the applicant was accused of incorrectly preparing a tax return. What was the accusation?” Options were “inadequate knowledge of tax codes,” “intentionally underreported a client’s capital gain,” and “neither of the above.” They were then asked, “what was the problem with this accusation?” Options included “primarily the applicant’s technical ability (i.e., understanding of tax codes),” “primarily the applicant’s integrity (i.e., willingness to bend the rules),” and “neither of the above.” The third question assessed whether the respondent recognized the reaction to the violation, admitted the mistake, and apologized.

Dependent Variables Measures. Following Mcknight et al. (1998), we differentiate trust into trusting intentions and trusting beliefs. We believe that responses to trust violations may influence trust beliefs and trust intentions. Two independent multi-item scales were used to assess trust beliefs in this study. Perceived integrity and perceived competence were measured using a three-part scale adapted by Mayer and Davis (1999) to assess participants’ perceptions of the applicant’s integrity and competence. Two additional scales were used to assess trust intention. The first was willingness to risk, measured by three items adapted by Mayer and Davis (1999) to capture the degree to which participants were willing to take risks in selecting candidates. Two of them were reverse scores. The second is whether to hire. We would capture participants’ intention to trust by their willingness to hire candidates.

Perceived integrity. We used three items to assess candidates’ integrity. The scale was also based on the integrity scale used by Mayer and Davis (1999). First, respondents were asked to rate the following items: (1) I really like the candidate’s values, (2) the applicant’s behavior meets norms, and (3) the applicant is honest, using a 5-point Likert scale (1 = “strongly disagree”; 5 = “strongly agree”).

Perceived competence. Similar to the Integrity Scale, we also used three items based on the Competency Scale of Mayer and Davis (1999) to assess the applicant’s competence. The items are as follows: (1) the applicant is capable of completing his/her work; (2) The applicant has sufficient knowledge required for the job; (3) I have great confidence in the applicant’s skills. In addition, respondents rated the questions on a 5-point Likert scale (1 = “strongly disagree” to 5 = “strongly agree”).

Willingness to risk. We used three items to measure participants’ willingness to risk, the degree to which they were willing to put themselves at risk in choosing a candidate. The items were as follows: (1) I will not let the applicant influence me on issues that are important to me (reverse-scored); (2) I will pay close attention to the applicant (reverse-scored); (3) I will give the applicant a task or problem that is important to me, even if I can’t monitor his/her actions. Respondents used the 5-point Likert Scale (1 = “strongly disagree”; 5 = “strongly agree more”). The scale was adapted from the trust scale used by Mayer and Davis (1999).

Hiring Intention. We asked participants to rate their willingness to hire a candidate on a 5-point Likert scale of “definitely not” and “definitely.” This indicator expresses trust intentions by whether or not to hire a candidate (Schwarz, 1990).

#### Results and Discussion

Manipulation checks revealed that the manipulations were successful. A total of 499 pieces of data were collected, in which 489 people answered the first question correctly, χ^2^ (2, *N* = 499) = 243.683, *p*-value < 0.001, and 490 people answered the second question correctly, χ^2^(2, *N* = 499) = 226.225, *p*-value < 0.001, The number of people who answered the third question correctly was 487, χ^2^(2, *N* = 499) = 921.768, *p*-value < 0.001. Confirmatory factor analyses of the trusting beliefs and trusting intentions variables (perceived competence, perceived integrity, willingness to risk, and hiring intention) indicated a good fit and supported convergent validity for a four-factor model, χ^2^(24, *N* = 499) = 63.05, GFI = 0.99, NFI = 0.99, RMSEA = 0.058, The factor coefficients of each item corresponding to the three variables of perceived integrity, perceived competence and Willingness to risk are all above 0.5, indicating that the corresponding item of each variable has certain representativeness. In addition, Average Variance Extracted (AVE) of perceived integrity, perceived ability, and willingness to risk were greater than 0.5, and Composite Reliability (CR) was greater than 0.7, indicating that the convergence validity of perceived integrity, perceived competence, and willingness to risk was ideal (*p-*value < 0.001).

Descriptive statistics, reliability, and correlation of study variables are shown in Table 1. Table 2 shows the mean and standard deviations of the variables for verbal and written apologies for violation of competence and integrity.

**TABLE 1:** Study 1 means, standard deviations, reliabilities, and intercorrelations.

Variable	*M*	SD	α	1	2	3
1. Perceived integrity	10.79	2.09	0.832	0.796		
2. Perceived competence	13.25	2.81	0.906	0.374**	0.885	
3. Willingness to risk	10.60	2.68	0.883	0.137*	0.113*	0.846

**p < 0.01, *p < 0.05.

**TABLE 2:** Study 1 means, standard deviations of verbal apology and written apology.

			Trusting beliefs				Trusting intentions			
			
			Perceived integrity		Perceived competence		Willingness to risk		Hiring	
Violation type	Apology type	*N*	*M*	SD	*M*	SD	*M*	SD	*M*	SD
Integrity	Verbal	124	10.18	1.87	12.40	2.91	10.10	2.46	2.73	1.09
Integrity	Written	122	11.52	2.00	13.83	2.99	11.19	2.77	3.54	1.09
Competence	Verbal	120	11.43	2.03	14.04	2.57	11.17	2.67	3.62	1.18
Competence	Written	121	10.03	2.05	12.78	2.44	9.98	2.59	2.78	1.13

Interaction items of violation type and apology type had significant positive effects on perceived integrity (β = 2.74, *p*-value < 0.001), perceived competence (β = 2.70, *p*-value < 0.001), and willingness to risk (β = 2.28, *p*-value < 0.001), supporting Hypothesis 1a.

**FIGURE 2:**
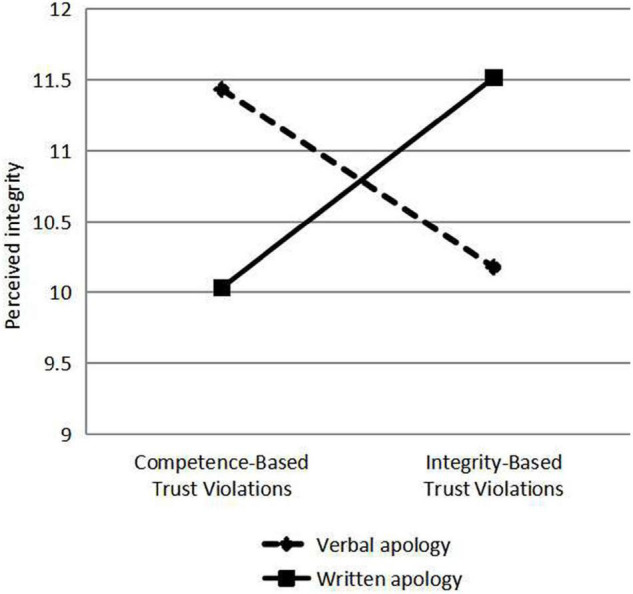
Effects of violation type and response on perceived integrity.

**FIGURE 3:**
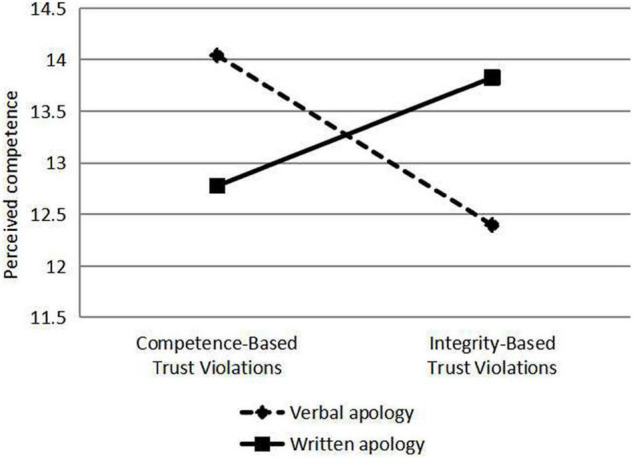
The effects of violation type and response on perceived competence.

**FIGURE 4:**
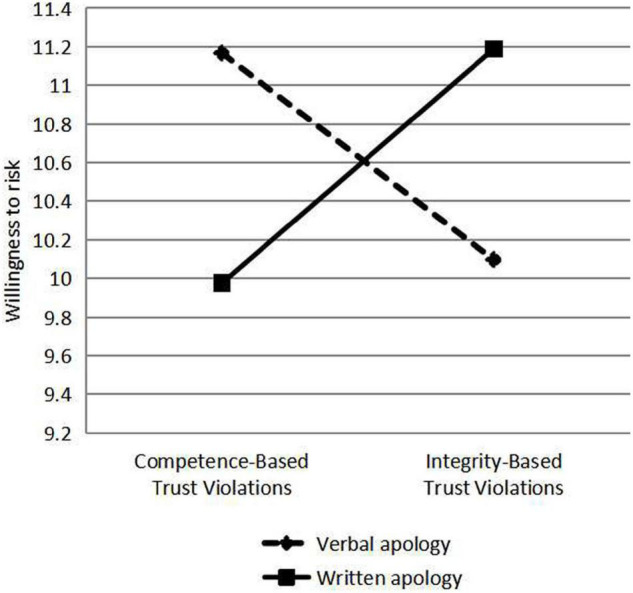
The effects of violation type and response on willingness to risk.

**FIGURE 5:**
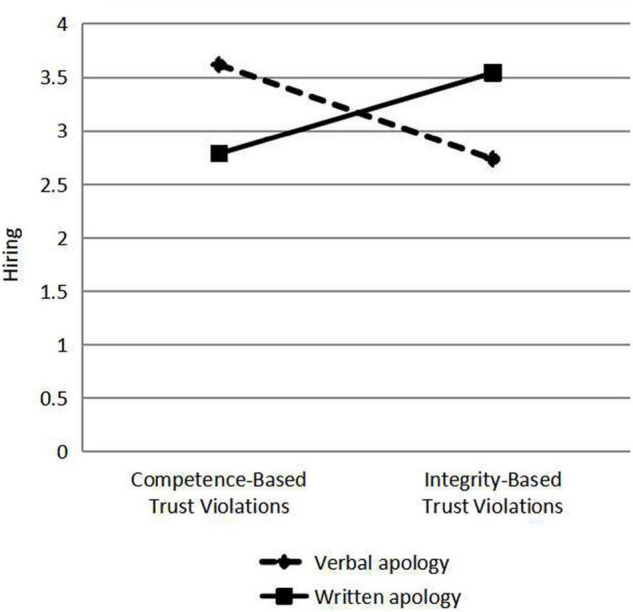
The effects of violation type and violation response on hiring.

Verbal and written apologies are responses to violations of trust. Different types of violation were classified according to the types of apologies, and the scores of perceived integrity, perceived competence, willingness to risk and hiring were analyzed by Analysis of Variance (ANOVA), and the differences of perceived integrity, perceived competence, willingness to risk and hiring under different types of violation, and apology were compared using ANOVA (see Figures 2–5). As a response to an integrity violation, written apology was significantly more effective than verbal apology in terms of its effects on perceived integrity (11.52 vs. 10.18; mean difference = 1.34, S.E. = 0.90), *p*-value < 0.001 (95% CI = [15.06, 18.60]), perceived competence (13.83 vs. 12.40; mean difference = 1.43, S.E. = 1.24), *p*-value < 0.001 (95% CI = [17.20,22.10]), and willingness to risk (11.19 vs. 10.10; mean difference = 1.09, S.E. = 1.19), *p*-value < 0.001 (95% CI = [13.36, 18.06]), and hiring intention (3.54 vs. 2.73; mean difference = 0.81, S.E. = 0.51), *p*-value < 0.001 (95% CI = [5.96, 7.98]). Verbal apologies are more effective as a response to competence violations than written ones because verbal apologies have a greater impact on perceived integrity (11.43 vs. 10.03; mean difference = 1.4, *p*-value < 0.001), perceived competence (14.04 vs. 12.78; mean difference = 1.26, *p*-value < 0.001), and willingness to risk (11.17 vs. 9.98; mean difference = 1.19, *p*-value < 0.001), and hiring intention (3.62 vs. 2.78; mean difference = 0.84, *p*-value < 0.001). When trust violation involves competence, trust is better repaired by responding with a verbal apology rather than a written one. When trust violation involves integrity, trust is better repaired by responding with a written apology rather than a verbal one. These analyses supported our Hypothesis 1b and 1c.

In experiment 1, participants who could not correctly understand the meaning of the experiment because they failed the attention check. Moreover, they are only a minimal number of respondents, and excluding them does not influence the experiment results. However, this study cannot perform a difference test between them due to limited omitted samples. Therefore, future research should split into two groups and conduct a comparative study between passed and failed the attention check if we have enough samples.

### Study 2

We conducted a scale survey to investigate the mediating effect of perceived credibility and repair.

#### Method

Participants. Three hundred thirty-five students in a marketing course at a university in eastern China took part in the study as a class assignment. The average age of the participants was 19.17 years (SD = 2.71), and 59% of them were male.

Task. The participants were divided into two groups based on violation of integrity or competence and then asked to play the role of a manager in recruiting and managing a senior tax accountant. Participants first read a written document, then took a test of perceived trustworthiness, and then evaluated the candidates.

Manipulations. The scale of perceived credibility was developed by Ohanian and had been extensively verified in studies (Ohanian, 1990, 1991). The scale included “Dependability, Honesty, Sincerity, and Trustworthy,” and respondents used the five-point Likert Scale to assess perceived credibility. Participants as managers of the recruitment process referenced study 1.

#### Results and Discussion

The mediating role of perceived credibility. This study performed meditation test by adopting Process (Hayes, 2017). The mediating effect of perceived credibility between trust violation and perceived integrity was significant (Effect = –2.86; 95% CI = [–3.51, –2.29]), and the mediating Effect between trust violation and perceived competence was significant (Effect = –2.58; 95% CI = [–3.21, –2.01]), and there was a significant mediating Effect between trust violation and willingness to risk (Effect = –2.18; 95% CI = [–2.77, –1.65]), and there was a significant mediating Effect between trust violation and hiring intention (Effect = –0.78; 95% CI = [–0.99, –0.59]). Therefore, Hypothesis 2a is confirmed.

The role of perceived credibility in different violation types. Different types of violation were classified according to the types of apology, and the scores of perceived integrity, perceived competence, willingness to risk and hiring were analyzed by ANOVA. Perceived credibility is more effective under competence violation than integrity violation, perceived integrity (11.10 vs. 6.97; mean difference = 4.13, *p*-value < 0.001), perceived competence (11.19 vs. 7.05; mean difference = 4.14, *p*-value < 0.001), and willingness to risk (11.51 vs. 7.80; mean difference = 3.71, *p*-value < 0.001), and hiring intention (3.78 vs. 2.56; mean difference = 1.22, *p*-value < 0.001). Hypothesis 2b is confirmed.

### Study 3

To explore the mediating role of positive emotions in trust violation and trust repair, we induced positive emotions through a direct writing task.

#### Method

Two hundred eighty-six students participated in a management course at a college in eastern China took part in the study as a classroom exercise. The average age of the participants was 19.14 years (SD = 1.82), and 65% were male.

Task Participants were divided into two groups based on integrity or competence violations and then asked to participate in an emotion-inducing task followed by completing an emotion-checking test. All respondents were asked to take on the role of a manager in recruiting and managing a senior tax accountant. Next, participants read a written statement and then evaluated the candidates.

Manipulations During the emotional induction, participants completed writing tasks designed to manipulate emotions. The emotive-eliciting program was developed by Strack et al. (1985), which was accomplished by direct writing. That method has been extensively validated in research (Kehner et al., 1993; Lerner and Keltner, 2001; Dunn and Schweitzer, 2005). The task asked participants to describe two or three things that made them feel really happy, at a level that would make someone else feel happy, to elicit positive emotions. Participants as managers of the recruitment process referenced study 1.

#### Results and Discussion

Emotional manipulation check. We also recruited forty-one students from the innovation and Entrepreneurship course to participate in the emotional manipulation check. In the manipulation check, firstly, participants were asked to describe things they felt happy about, in the sense that others would also feel happy about them, and then rated the extent of their current emotional experience on a 5-point Likert scale ranging from 1 “very unhappy” to 5 “very happy.” The results showed that positive emotions were successfully induced (*M* = 4.10, SD = 0.14).

The moderating role of positive emotions. To test the moderation effect, this study conducted ANOVA. The empirical results showed that interaction items of violation type and positive emotion had significant positive effects on perceived integrity (β = 0.63, *p*-value < 0.001), perceived competence (β = 1.51, *p*-value < 0.001), and willingness to risk (β = 0.70, *p*-value < 0.001), and hiring intention (β = 0.77, *p*-value < 0.001), supporting Hypothesis 3a. These results suggest that positive emotions play a partially moderating role in the relationship between trust violation and trust repair. Hypothesis 3a is confirmed.

The role of positive emotion in different types of disobedience. Positive emotions are more effective under integrity violations than competence violations, perceived integrity (11.97 vs. 7.78; mean difference = 4.19, *p*-value < 0.001), perceived competence (11.61 vs. 8.09; mean difference = 3.52, *p*-value < 0.001), and willingness to risk (11.95 vs. 9.08; mean difference = 2.87, *p*-value < 0.001), and hiring intention (3.98 vs. 2.92; mean difference = 1.06, *p*-value < 0.001).

## General Discussion, Implications, and Limitations and Feature Research

### General Discussion

Whether to trust someone or not is very challenging (Perrone et al., 2003). When trust is broken, it is difficult to trust. But violations of trust, whether intentional or unintentional, are common. Researchers must therefore study how trust is established and how individuals react when they perceive that trust has been violated. In general, the study of this reaction is still in its infancy (Kim et al., 2004, 2006).

This study aimed to investigate the effects of a verbal apology and a written apology on trust repair after trust violations based on competence and integrity and to investigate the mediating role of perceived credibility and the moderating role of positive emotions in both types of trust violations. Firstly, consistent with our predictions, we found that verbal and written apologies effectively restored trust. However, verbal apologies were more effective than written apologies under competency-based trust violations, and under integrity-based trust violations, a written apology is more effective than a verbal one. Secondly, our study has revealed that perceived credibility mediates between trust violation and trust repair and is more effective under competence-based violations than integrity-based violations. Thirdly, we also examined the moderating role of positive emotions between trust violations and trust restoration, and the effect was higher under integrity-based violations than competence-based violations.

### Theoretical Contributions

Our research investigates how trust is repaired after a breach, and while previous investigations in this area have concluded that many repair strategies (apology, explanation, compensation) contribute to effective trust repair, this paper extends these insights in three ways. (1) This study divides apology into verbal apology and written apology, respectively, revealing its effectiveness in trust restoration, and which apology form is more effective depends on the type of trust violation; (2) The role of perceived credibility is investigated; (3) Positive emotion variables is also introduced, and its effectiveness is analyzed.

Firstly, existing studies have confirmed the positive effect of apology on repairing trust. Kim et al. (2006) divided apology into internal attribution and external attribution, believing that whether apology is effective depends on the type of trust violation. This study expands on these ideas by exploring the relationship between apology and types of aggression. An apology can be divided into verbal apology and written apology. Based on the types of trust breach, it is concluded that verbal apology has a better effect than written apology under competence-based trust breach, and in the case of integrity-based trust breach, a written apology is more effective than a verbal apology.

Secondly, this study explores the relationship between perceived credibility in violation and trust repair, further expanding people’s understanding of perceived credibility in the field of trust, and conducts classified research on different types of violation, enriching the mechanism of perceived credibility.

Thirdly, current research confirms the positive effect of positive emotions on trust (Lount, 2010) and a moderating role between trust repair strategies and trust repair. However, no studies have used positive emotions as a moderator between trust violation and trust repair. This study found that positive emotion can play a moderating role between trust violation and trust restoration and found that positive emotion has different effects between different types of trust violations, and the influence of integrity violations is higher than that of competence violations. These studies extend the application of positive emotions in trust repair and further explain the relationship between trust violation and repair from an emotional perspective.

### Managerial Implications

The studies also highlight some issues that may need management attention. First of all, the results are helpful for enterprises or individuals to choose more effective forms of apology to repair trust after a trust breach. When the breach of trust involves competence, a verbal apology that can demonstrate competence is more appropriate. When a violation of trust is a matter of integrity, a more formal and sincere written apology is more likely to solve the problem. Secondly, the effect of perceived credibility of merchants or individuals on trust repair cannot be ignored. Merchants should make efforts to improve perceived credibility so as to lay a foundation for trust repair. Thirdly, positive emotions can promote the repair of consumers’ trust. The enlightenment of this conclusion for businesses is that when repairing the trust relationship between the two sides, businesses should actively identify consumers’ emotions and even actively create a good emotional atmosphere to give full play to the role of positive emotions in promoting trust.

### Limitations and Future Research

This study has some limitations and provides a direction for further research. In this study, situational experiments were used to evaluate consumers’ perception of the types of trust violation and the effect of trust repair. Although situational experiments have obvious advantages in marketing research, how to create a better scenario for surfeiters remains a problem. Field experiments, reading materials, and questionnaires have certain limitations and cannot completely restore the environment. Second, the survey subjects are all college students who generally lack recruitment experience and do not fully understand the work of the accountants involved in the study, so expanding the demographic range of survey participants may yield rich insights. Third, we should note that the results of this study cannot prove whether the apology is enough to restore trust to the level before the violation completely, and trust measures before and after the violation need to be obtained. Subsequent studies will overcome this problem and try to solve the difficult problem of whether trust can be “completely repaired.” Fourth, response time for trust repair is also critical (Wirtz and Mattila, 2004). This study did not explore the use of “immediate” in the trust repair process to reduce the interference of response time on trust repair, and future research needs to explore the impact of response time and interaction with other factors on trust repair. Fifth, the hypotheses of this paper are limited to the violation and repair of personal trust, and its applicability to enterprises, media and countries needs further discussion and research. Sixth, although there is an intrinsic relationship between the three studies, there are still some substitutability, and the intrinsic relationship between apology and positive emotions and perceived credibility should be further sorted out in future research. Seventh, For the assessment of mood, no baseline was collected, which can be used in future studies to better show changes through comparison. Eighth, in study 1, we found that verbal apology has a greater impact on perceived integrity, which seems to contradict Hypothesis 1c, although the hypothesis holds in general, and we will test it with more research in future research. The last, Kim et al. applied SEM to analyze important factors for trust in human-robot interaction. Structural equations can solve more complex models with deeper and more reliable results. In future research, we can try to use Structural Equations Modeling to study the relationship between more variables about trust restoration (Kim et al., 2020).

## Data Availability Statement

The raw data supporting the conclusions of this article will be made available by the authors, without undue reservation.

## Ethics Statement

Ethical review and approval was not required for the study on human participants in accordance with the local legislation and institutional requirements. Written informed consent from the patients/participants was not required to participate in this study in accordance with the national legislation and the institutional requirements.

## Author Contributions

SG and JY: conceptualization, methodology, validation, data curation, and writing—review and editing. SG: formal analysis, writing—original draft preparation, and supervision. JY: visualization and project administration. Both authors have read and agreed to the published version of the manuscript.

## Conflict of Interest

The authors declare that the research was conducted in the absence of any commercial or financial relationships that could be construed as a potential conflict of interest.

## Publisher’s Note

All claims expressed in this article are solely those of the authors and do not necessarily represent those of their affiliated organizations, or those of the publisher, the editors and the reviewers. Any product that may be evaluated in this article, or claim that may be made by its manufacturer, is not guaranteed or endorsed by the publisher.
